# Response of phytoplankton to protective-restoration treatments enhancing water quality in a shallow urban lake

**DOI:** 10.1007/s10661-016-5633-4

**Published:** 2016-10-18

**Authors:** Elżbieta Zębek, Agnieszka Napiórkowska-Krzebietke

**Affiliations:** 1 Faculty of Law and Administration, Department of International Public Law, University of Warmia and Mazury, Warszawska 98, 10-702 Olsztyn, Poland; 2 Department of Hydrobiology, Inland Fisheries Institute in Olsztyn, Oczapowskiego 10, 10-719 Olsztyn, Poland

**Keywords:** Urban lake, Restorative procedures, Phytoplankton, Water quality, RDA, CCA

## Abstract

Lake Jeziorak Mały is a shallow urban lake where storm water pretreatment separators and a fountain-based water aeration system were installed as protective-restoration measures to enhance water quality. We investigated the effect of these procedures on phytoplankton dynamics and physicochemical properties in the littoral and pelagial zones in 1996–2003, 2005, and 2013. A decrease in cyanobacteria proportion, abundance, and biomass has been noticed, and other phytoplankton groups increased after these procedures. Significantly elevated species diversity was recorded in the littoral zone with the exchange of cyanobacteria and diatom dominant species typically induced by alteration from hypertrophic to eutrophic status. For example, the polytrophic *Limnothrix redekei* was replaced by eutrophic *Planktolyngbya brevicellularis*. This stemmed from greater oxygenation, water visibility and diminished pH, conductivity, and orthophosphates. Our results showed that introducing these restoration measures influence on the long-term succession of phytoplankton and induced the change from a polytrophic to eutrophic state, and that such measures are vitally important in future considerations of shallow urban lake management.

## Introduction

Intensified urban industrial development contribute to water eutrophication (Bernhardt 1987; Reynolds 2003), which accelerates in urban agglomerations when domestic sewage and nutrient-rich stormwater enter shallow lakes (Guzkowska and Gasse 1990; Wichelen et al. 2007). Excess primary production causes massive growth of some algal species, particularly cyanobacteria, resulting in algal blooms and deterioration in water quality (Reynolds 1978; Spodniewska 1986; Bucka 1989; Napiórkowska-Krzebietke 2015; Napiórkowska-Krzebietke and Dunalska 2015; Napiórkowska-Krzebietke et al. 2015). These processes are effectively limited by reducing nutrient inflow, especially that of phosphorus, using lake-basin restoration methods (Bernhardt 1987; Reynolds 2003), including external protective methods in the lake’s catchment area (Lossow 1998; Dunalska et al. 2015), although it is quite difficult to establish the most suitable and permanently effective restoration methods for individual lakes. For effective protection of a lake, selection of the restoration method should consider the lake’s category (morphological and hydrological conditions), pollution sources, manners of the catchment’s management, and technical possibilities relevant for local conditions (Bernhardt 1987). In urbanized catchments without separate sewage system for rainwater and sewage, polluted storm waters are often discharged directly into the lake (Guzkowska and Gasse 1990). In order to protect waters of urban lakes, separators are used for storm water pretreatment from polycyclic aromatic hydrocarbons (PAHs).

Phytoplankton is good indicator of lake trophic states and changes caused by nutrient inflow. It is evidenced by their role in the functioning of water ecosystems. Ecologically, phytoplankton plays an important role in nutrient cycling and biological productivity in aquatic system, linking a number of bottom-up and top-down processes (Reynolds 1984).

Lake Jeziorak Mały is an example of a eutrophic lake dominated by cyanobacteria in the phytoplankton, where protective-restoration measures were applied to improve water quality. This included installation of separators for storm water pretreatment and fountain-based aeration. The relevant hypothesis is that these restoration measures affect the long-term dynamics of phytoplankton via altered water quality. The purpose of this study was to determine the response of phytoplankton related to physicochemical properties to these restorative procedures in urban Lake Jeziorak Mały in1996–2003, 2005, and 2013.

## Material and methods

### Study area

**Fig. 1:**
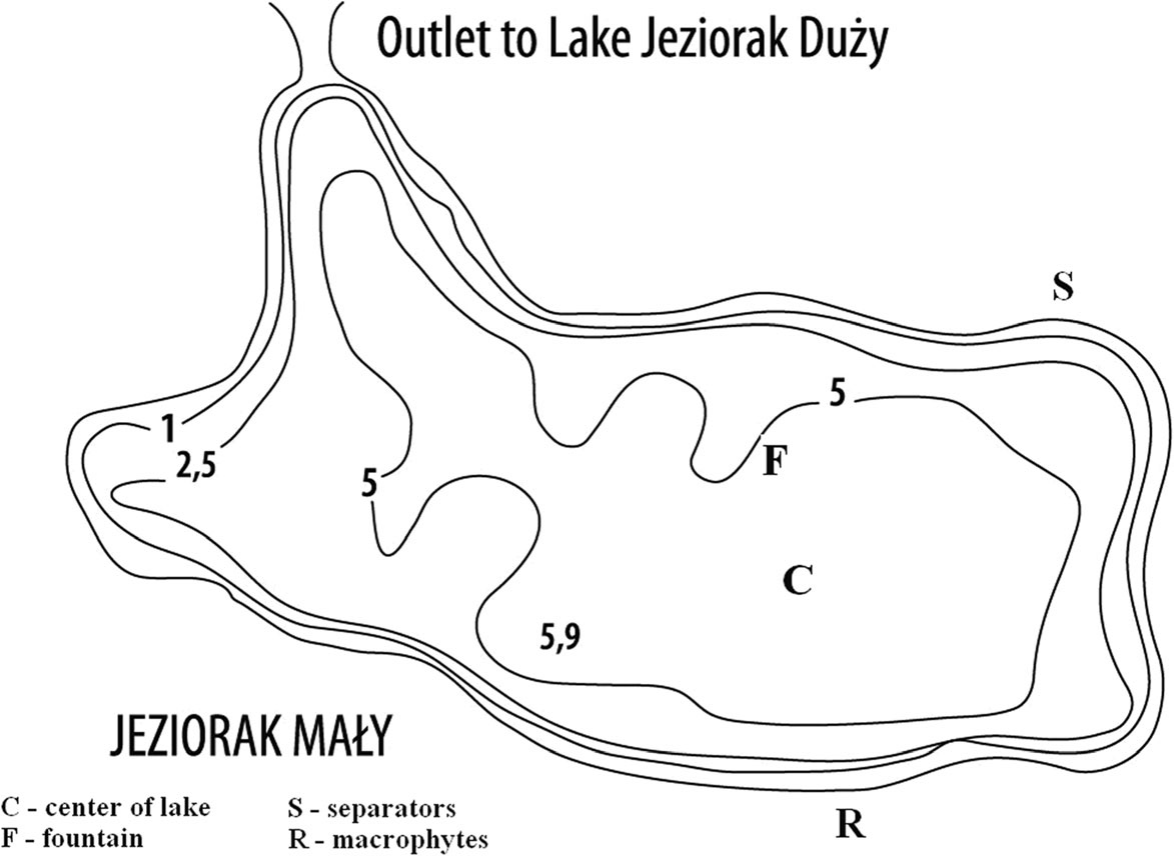
Morphometric map of Lake Jeziorak Mały

Lake Jeziorak Mały is a typical, shallow eutrophic urban lake with mean depth of 3.4 m in the Mazurian Lakeland of northeastern Poland. The 26-ha lake is situated in a temperate climatic zone. It is connected to the 3219 ha Lake Jeziorak Duży by a narrow canal 4 m wide and 4 m deep. Their connection consists of a concrete barrier for water leveling, to prevent mixing of waters from these two lakes with such diverse surface areas (Fig. 1). This shallow water body is subjected to continuous mixing with the very high (Giziński 1978) mixing coefficient of 0.9 (Zębek 2009).

For many decades, this lake received untreated municipal sewage from the town of Iława. Since 1991, however, effluent has been treated at a local wastewater treatment plant. Work to improve the lake’s water quality began in 1997 and remains ongoing, including installation of separators for pretreatment of stormwater discharged to the littoral zone and a fountain-based water aeration system in the pelagial. The lake forms a storm sewer system outlet, typical in smaller cities. Construction commenced in 1996 on the Unicon System lamella separators in the lake’s littoral zone, and their completion in spring 1997 prevented untreated stormwater transfer to the lake. These separators contained 16 blinder sections, 1200-mm diameter inlet and outlet pipes, and a 3200-L sedimentation tank to remove petroleum compounds, silt, and sand in the separate rainwater sewer system. The separation efficiency of petroleum derivatives was 97 % at a nominal discharge of 160 L s^−1^. The maximum separator discharge was 1600 L s^−1^, in which only 10 % of these substances were treated. This stormwater pretreatment covered the 70-ha catchment area of Lake Jeziorak Mały (PUH EKOL 1995). The lake shores are partly covered with concrete or reinforced with fascine, and most of the lake bed is stones and gravel. Lake Jeziorak Mały is an example of a reversed littoral zone management system, with approximately 30 % macrophytes and 70 % concrete bank. The phytolittoral includes emerged macrophytes, mainly *Phragmites australis* (Cav.) Trin. ex Steud., *Scirpus lacustris* (L.) Palla, *Acorus calamus* L., and *Glyceria aquatica* (L.) Wahlb., and the bed is muddy and covered with decomposing plant debris.

The fountain covered both the shoreline and the deeper part of the lake, and subsequent aeration transformed de-oxidized waters from the bottom layer of the 5-m deep lake bed to oxygenated water by shooting it 16 m above the surface and then spraying it over a 5-m lake cascade area. This results in aeration being limited in the superficial layer to 1-m depth. The fountain is driven by a SP 60-6 deep pump with 11-KWh power and maximum flow rate of 80 m^3^ h^−1^ (21 L s^−1^), with a water output volume of 0.14 m^3^. The rate is therefore 1 m^3^ every 45 s. This provides 80 % pump efficiency, with water stream maximum efficiency of 55 m^3^ h^−1^ or 15 L s^−1^ at 80-kPa pressure (GRUNDFOS 1997). The waters should be artificially aerated by 11,136 h (about 464 days) to pour the whole volume of lake. The advantage gained from this artificial aeration and increased water mixing was physicochemical changes in temperature, oxygenation, and nutrient concentrations.

### Phytoplankton and water sample collection

Phytoplankton samples were collected:

1. Monthly from April to October in 1996–2003, 2005, and 2013 at sites with separator pipe outlets draining stormwater (S), and at sites with macrophytes (R) in the littoral zone
2. Monthly from June to August in 1996–2003 and 2005 in the pelagial zone

The samples were taken from euphotic zone with a 10-L calibrated bucket (20 L at each site), sieved through a plankton net no. 25 and preserved with a Lugol’s solution and then with a 4 % formaldehyde solution. In total, 320 phytoplankton samples were tested.

The following physicochemical properties were measured directly at the phytoplankton sampling sites:

- Water temperature with 0.1 °C precision and oxygen content exact to 0.01 mg O_2_ L^−1^ (HI 9143 oxygen meter) in the water column from the surface to a depth of 4 m in the pelagial
- pH and electrolytic conductivity at 1–1500 μS cm^−1^ (CONMET 1)
- Visibility (Secchi disk)

### Phytoplankton and water samples analysis

The following groups of phytoplankton were analyzed in this study: cyanobacteria, diatoms, chlorophytes, dinoflagellates, chrysophytes, and cryptomonads. Qualitative and quantitative determinations of phytoplankton were performed with an Alphaphot YS2 optical microscope at magnifications of ×100, ×200, ×400, and ×1000. Numbers in 1 mL samples of phytoplankton were determined in 5000 fields of vision with ×200 magnification in each planktonic chamber to account for differences in organism densities and their abundance and biomass expressed in identical basic 1-mL volumes. Diatoms were prepared following the standard method in Battarbee (1979). Algal biomass for ten individuals was calculated by comparing algae with their geometric shape (Rott 1981).

The scope of water analysis in the laboratory included

- Orthophosphates (0.05–5.00 mg P-PO_4_ L^−1^ )
- TN (0.5–15 mg N L^−1^)
- Chlorides (2.5–250 mg Cl L^−1^)

using Spectroquant Merck tests with NOVA 400 spectrophotometer.

### Statistical analysis

In the statistical analysis, means were applied as the average values of phytoplankton abundance or biomass from the growth season (from April to October) in the littoral zone at stations with separators and with macrophytes and summer period (from June to August) in the pelagial zone in particular years of this study. The mean values of water physicochemical properties were calculated in the same way. The standard deviations and significance of differences between analyzed data (Mann-Whitney *U* test) were calculated with STATISTICA version 8. The conducted analysis demonstrated statistically significant differences between abundance and biomass of phytoplankton and physicochemical properties at stations at the separators and macrophytes in the littoral and pelagial zones at *p* < 0.05. The species diversity for phytoplankton abundance was analyzed to calculate the Shannon-Weaver index (Shannon and Weaver 1949).

Phytoplankton groups and dominant species abundance were correlated with physical and chemical water parameters using nonparametric methods because these data are not normally distributed. To reduce the number of variables, a forward selection procedure using the Monte Carlo test with 999 permutations. Relationships were confirmed by calculating Spearman’s rank correlation coefficient (*p* < 0.05) with STATISTICA version 8, and then with canonical correspondence analysis CCA to relate water chemistry variables to phytoplankton groups in the studied years, and RDA to determine the correlation between dominant species and physicochemical properties. Finally, these relationships were presented on a biplots graph using Canoco for Windows 4.5 software.

## Results

### Physicochemical properties

Differences in physicochemical properties were recorded between littoral sites with separators (S) and those with macrophytes (R) in 1996–2003, 2005, and 2013 (Table 1). Comparison revealed significant water temperature reduction at (S) locations from 13.5 °C in 2000 to 18.6 °C in 2003, while (R) sites ranged from 17.4 to 20.5 °C. Higher oxygenation was also noted at 6.93 mg O_2_ L^−1^ in 2002 and 9.26 mg O_2_ L^−1^ in 2003 (S), with pH varying from 9.35 in 1998 to 7.76 in 2013. The highest conductivity of 843 μS cm^−1^ in 1998 was also registered at S with its 332 μS cm^−1^ minimum in 2013. Similarly, there were higher orthophosphate, total nitrogen, and chloride concentrations at the separators. Orthophosphates measured 0.68 mg L^−1^ in 2000 (S) compared to 0.50 mg L^−1^ in 2005 (R), before declining to its lowest 0.11 mg L^−1^ in 2013. While TN ranged from 1.4 to 4.3 mg L^−1^, maximum Cl was 97 mg L^−1^ (S) in 2000 compared to the 47 mg L^−1^ (R) level achieved in 2001.

**Table 1:** Physical and chemical water parameters (mean and standard deviation M ± SD) in the littoral zone (April–October) and in the pelagial (June–August, surface layer (s), bottom (b), no data (–)) of urban Lake Jeziorak Mały in 1996–2003, 2005, and 2013

	1996	1997	1998	1999	2000	2001	2002	2003	2005	2013
Separators										
Water temperature (°C)	13.6 ± 3.46	16.9 ± 3.50	15.9 ± 2.34	16.8 ± 3.86	13.5 ± 2.29	18.8 ± 5.60	17.3 ± 3.63	18.6 ± 3.78	17.1 ± 4.19	18.0 ± 4.85
Oxygen (mg L^−1^)	8.30 ± 2.86	8.17 ± 2.71	6.90 ± 0.97	9.03 ± 1.63	8.14 ± 1.26	8.51 ± 2.19	6.93 ± 3.40	9.26 ± 1.84	8.38 ± 4.41	8.42 ± 3.63
pH	8.82 ± 1.26	8.74 ± 0.87	9.35 ± 0.91	8.49 ± 1.33	8.28 ± 0.59	7.93 ± 0.46	9.14 ± 1.21	8.63 ± 0.75	9.51 ± 1.44	7.76 ± 0.45
Conductivity (μS cm^−1^)	852 ± 345	757 ± 387	843 ± 165	567 ± 259	744 ± 233	457 ± 100	418 ± 218	346 ± 52	459 ± 234	332 ± 59
P-PO_4_ (mg L^−1^)	0.46 ± 0.65	0.45 ± 0.68	0.32 ± 0.20	0.50 ± 0.47	0.68 ± 0.48	0.13 ± 0.11	0.29 ± 0.34	0.44 ± 0.41	0.47 ± 0.08	0.11 ± 0.05
TN (mg L^−1^)	–	–	–	1.4 ± 0.6	2.9 ± 1.1	1.6 ± 0.9	3.0 ± 1.2	4.3 ± 1.7	3.4 ± 3.1	3.2 ± 2.9
Cl (mg L^−1^)	40 ± 14	66 ± 22	68 ± 25	30 ± 18	97 ± 28	39 ± 33	33 ± 12	54 ± 19	–	–
Macrophytes										
Water temperature (°C)	17.7 ± 2.76	18.8 ± 4.80	19.0 ± 4.73	20.0 ± 7.72	17.4 ± 5.33	19.2 ± 5.74	19.3 ± 5.42	20.5 ± 4.11	19.1 ± 4.68	20.3 ± 3.89
Oxygen (mg L^−1^)	7.40 ± 3.80	9.65 ± 5.11	7.35 ± 2.59	7.90 ± 2.82	7.35 ± 2.85	8.26 ± 2.71	5.63 ± 2.59	10.27 ± 5.48	8.05 ± 1.99	8.69 ± 2.12
pH	9.92 ± 0.55	9.01 ± 0.50	9.93 ± 0.84	8.73 ± 0.61	8.74 ± 0.44	8.42 ± 0.32	9.08 ± 1.16	8.61 ± 0.68	8.97 ± 1.13	7.70 ± 0.78
Conductivity (μS cm^−1^)	415 ± 135	394 ± 129	418 ± 76	387 ± 68	486 ± 163	423 ± 203	446 ± 318	322 ± 37	665 ± 538	323 ± 67
P-PO_4_ (mg L^−1^)	0.32 ± 0.26	0.20 ± 0.17	0.05 ± 0.03	0.21 ± 0.28	0.17 ± 0.07	0.11 ± 0.19	0.31 ± 0.34	0.39 ± 0.38	0.50 ± 0.05	0.44 ± 0.42
TN (mg L^−1^)	–	–	–	1.0 ± 0.4	2.5 ± 0.9	2.2 ± 0.8	3.0 ± 1.2	1.8 ± 1.1	3.0 ± 1.5	4.1 ± 2.1
Cl (mg L^−1^)	25 ± 11	20 ± 4	26 ± 4	25 ± 5	32 ± 8	47 ± 12	28 ± 11	36 ± 12	–	–
Pelagial										
Water temperature (°C)	18.6 ± 2.86	25.1 ± 3.32	20.6 ± 1.92	27.0 ± 2.05	20.3 ± 0.46	24.1 ± 1.25	23.3 ± 1.37	23.9 ± 3.14	25.6 ± 2.83	–
Oxygen (mg L^−1^) (s-b)	9.22 ± 3.67 −0	10.05 ± 4.40 −0	10.73 ± 2.62 −1.01 ± 0.04	10.63 ± 0.89 −3.65 ± 0.80	10.85 ± 0.60 −0	9.50 ± 1.06 −0	12.15 ± 2.20 −0	9.60 ± 2.25 −0.53 ± 0.04	10.49 ± 1.67 −0	–
pH	10.02 ± 0.25	10.06 ± 0.27	10.20 ± 0.26	9.52 ± 0.29	9.13 ± 0.25	9.23 ± 0.39	8.60 ± 0.43	8.63 ± 0.61	8.41 ± 1.07	–
Conductivity (μS cm^−1^)	332 ± 114	324 ± 125	326 ± 12	333 ± 32	353 ± 83	328 ± 17	272 ± 28	272 ± 38	308 ± 18	–
P-PO_4_ (mg L^−1^) (s-b)	0.05 ± 0.07 −2.43 ± 1.89	0.04 ± 0.08 −2.42 ± 1.90	0.03 ± 0.04 −1.29 ± 0.82	0.06 ± 0.03 −1.25 ± 0.60	0.08 ± 0.07 −0.26 ± 0.05	0.01 ± 0.03 −1.15 ± 0.07	0.17 ± 0.03 −0.45 ± 0.05	0.16 ± 0.06 −0.50 ± 0.24	0.36 ± 0.12 −0.52 ± 0.32	–
TN (mg L^−1^) (s-b)	–	–	–	0.5 ± 0.7 −2.8 ± 0.9	–	–	0.5 ± 0.3 −2.9 ± 1.4	1.4 ± 0.7 −7.3 ± 3.2	2.7 ± 2.0 −5.5 ± 3.5	–
Cl (mg L^−1^)	23 ± 12	21 ± 8	24 ± 9	5 ± 2	25 ± 8	23 ± 7	22 ± 3	27 ± 2	25 ± 3	–
Water visibility (m)	0.35 ± 0.11	0.50 ± 0.21	0.65 ± 0.23	0.40 ± 0.15	0.75 ± 0.25	0.65 ± 0.21	0.90 ± 0.32	1.20 ± 0.33	0.70 ± 0.23	0.80 ± 0.24

Although pelagial physicochemical properties varied in the 1996–2003 and 2005 summer months, with clearer water transparency and oxygenation rising from 0.35 m in 1996 to 1.20 m in 2003, and from 9.22 mg O_2_ L^−1^ in 1996 to 12.15 mg O_2_ L^−1^ in 2002, there were oxygen deficits at the lake bed. In contrast, pH in this period fell from10.06 to 8.33, water conductivity from 324 to 277 μS cm^−1^, orthophosphates from 2.24 to 0.45 mg L^−1^, and chlorides increased from 5 to 27 mg L^−1^.

### Phytoplankton in the littoral zone

**Fig. 2:**
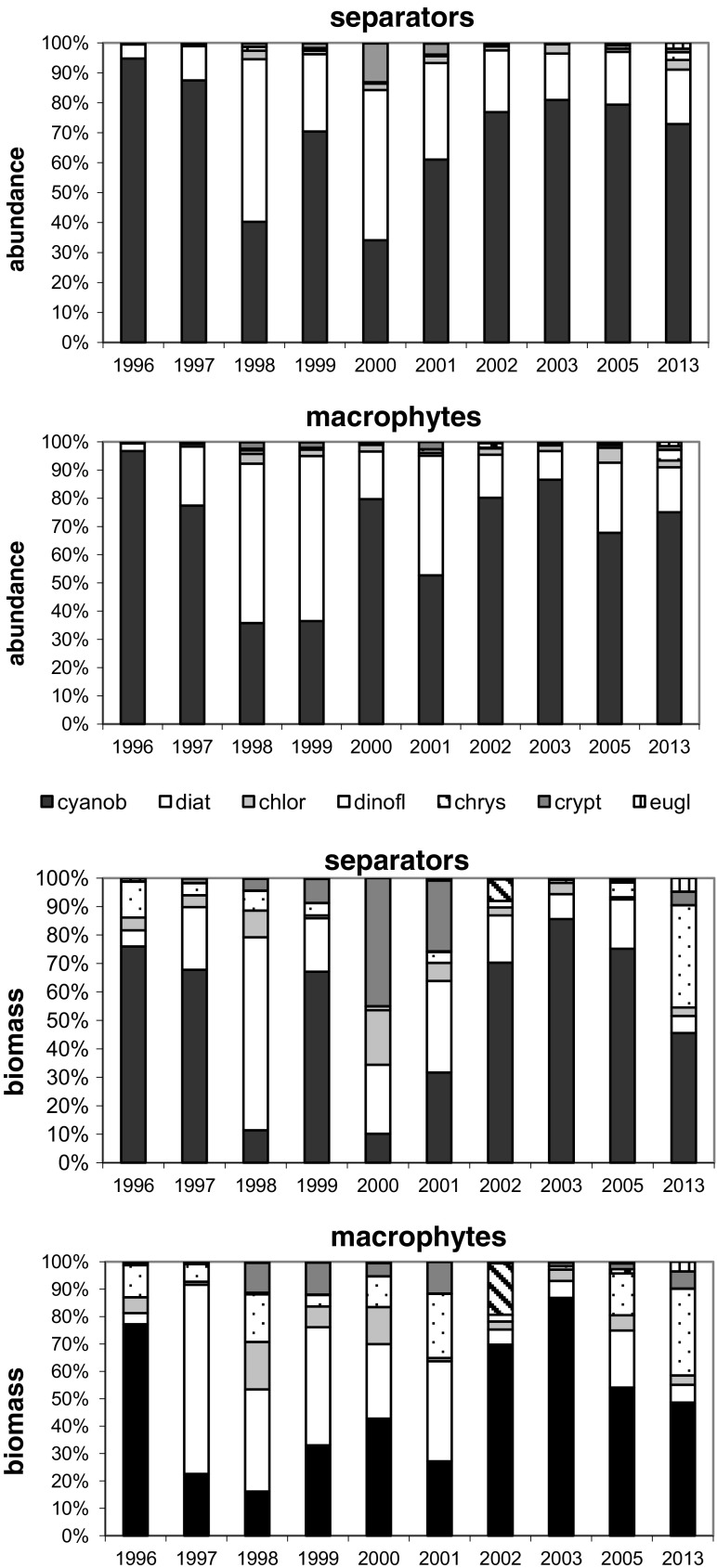
Structure of phytoplankton (*cyanob* cyanobacteria, *diat* diatoms, *chlor* chlorophytes, *dinofl* dinoflagellates, *chrys* chrysophytes, *crypt* cryptomonads, *eugl* euglenines) in the littoral zone of Lake Jeziorak Mały in 1996–2003, 2005, and 2013

Phytoplankton abundance and biomass were dominated by cyanobacteria in the littoral zone, with lower algal proportion at the separators (S) than at sites with macrophytes (R). The highest proportion of cyanobacteria in total phytoplankton abundance was 94.77 % (S) and 96.74 % (R) in 1996, followed by significant decrease to 34.15 % (S) in 2000 and 36.49 % (R) in 1998–1999. Their percentage then rose during 2001–2003 and 2002–2003 to final 2013 composition of 72.91 % (S) and 75.07 % (R). While, diatoms and cryptomonads shared significant percentages of total phytoplankton abundance; with diatom levels at 54.24 % (S) in 1998 and 58.48 % (R) in 1999 and cryptomonads at 12.97 % (S) in 2000, and chlorophytes were relegated to their ultimate 5.25 % (R) in 2005. Hence, remaining phytoplankton groups did not exceed 5 %. Cyanobacterial percentages of phytoplankton biomass mirrored abundance. Here, (S) initially decreased dramatically from 76 % in 1996 to 10.14 % in 2000 but recovered to 45.58 % in 2013 and (R) plummeted from 77.21 % in 1996 to 16.12 % in 1998 before finally achieving 48.62 % in 2013. After 1996, increased proportions of remaining phytoplankton groups in the total phytoplankton biomass. These included attached (S) diatom maximum of 67.82 % in 1998 with 45 % cryptomonads and 19.18 % chlorophytes in 2000 and also 35.90 % dinoflagellates in 2013, while (R) chrysophytes registered 18.77 % in 2002 (Fig. 2).

**Fig. 3:**
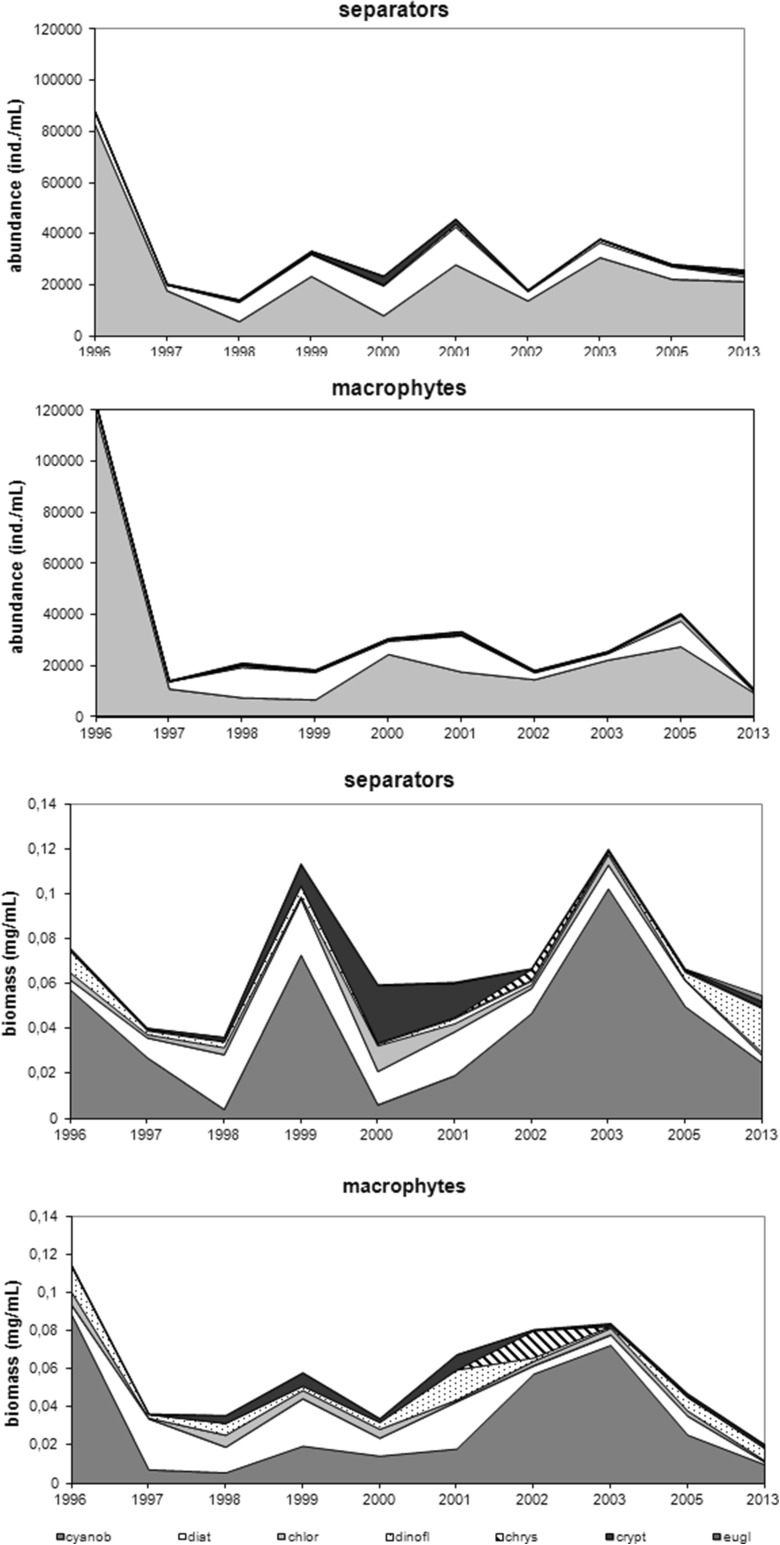
Dynamics of abundance and biomass of phytoplankton (*cyanob* cyanobacteria, *diat* diatoms, *chlor* chlorophytes, *dinofl* dinoflagellates, *chrys* chrysophytes, *crypt* cryptomonads, *eugl* euglenines) in the littoral zone of Lake Jeziorak Mały (means from April to October in 1996–2003, 2005, and 2013)

Maximum cyanobacteria abundances in the littoral zone were recorded in 1996 at 83,390 ind. mL^−1^ (S) and 118,390 ind. mL^−1^ (R). This was followed by inevitable rapid declines in 1997 and 1998, with recoveries in 1999, 2000, and 2003 to final 2013 levels of 21,430 ind. mL^−1^ (S) and 9560 ind. mL^−1^ (R). Here, cyanobacteria abundance reduction was accompanied by increased diatom levels to14,780 ind. mL^−1^ (S) and 14,250 ind. mL^−1^ (R) in 2001 and also chlorophyte, and cryptomonad escalation especially at (S) sites. Biomass levels also fluctuated, with significant loss of cyanobacterial biomass to 0.0041 mg mL^−1^ (S) and 0.0058 mg mL^−1^ (R) in 1997–1998, followed by compensation between 1998 and 2003 to 0.1026 mg mL^−1^ maximum in 2005 before final relapse to 0.0249 mg mL^−1^ in 2013. Maximum diatom biomass was noted in 1999 at both sites, where (S) cryptomonads, chlorophytes, and dinoflagellates increased and (R) dinoflagellates and chrysophytes were also prominent (Fig. 3).

Statistically significant relationships were established between phytoplankton group abundance and physicochemical properties (*N* = 50, *p* < 0.05). Here, (S) cyanobacteria, dinoflagellates, and chrysophytes positively correlated with water temperature (*r* = 0.62, *r* = 0.48, *r* = 0.32, respectively) and diatoms with oxygen content (*r* = 0.42). In contrast, negative correlations were recorded between cyanobacteria and conductivity (*r* = −0.42), and between chrysophytes, cryptomonads, euglenin, and P-PO_4_ (with *r* = −0.31, *r* = −0.29, and *r* = −0.33). In addition, (R) cyanobacteria and dinoflagellates positively associated with water temperature (*r* = 0.41 and 0.51), diatoms with oxygen content (*r* = 0.29), and diatoms, chlorophytes, and cryptomonads with conductivity (*r* = 0.37, *r* = 0.43, and *r* = 0.44, respectively). Diatoms and chlorophytes, however, negatively correlated with water temperature (*r* = −0.43 and *r* = −0.38).

**Fig. 4:**
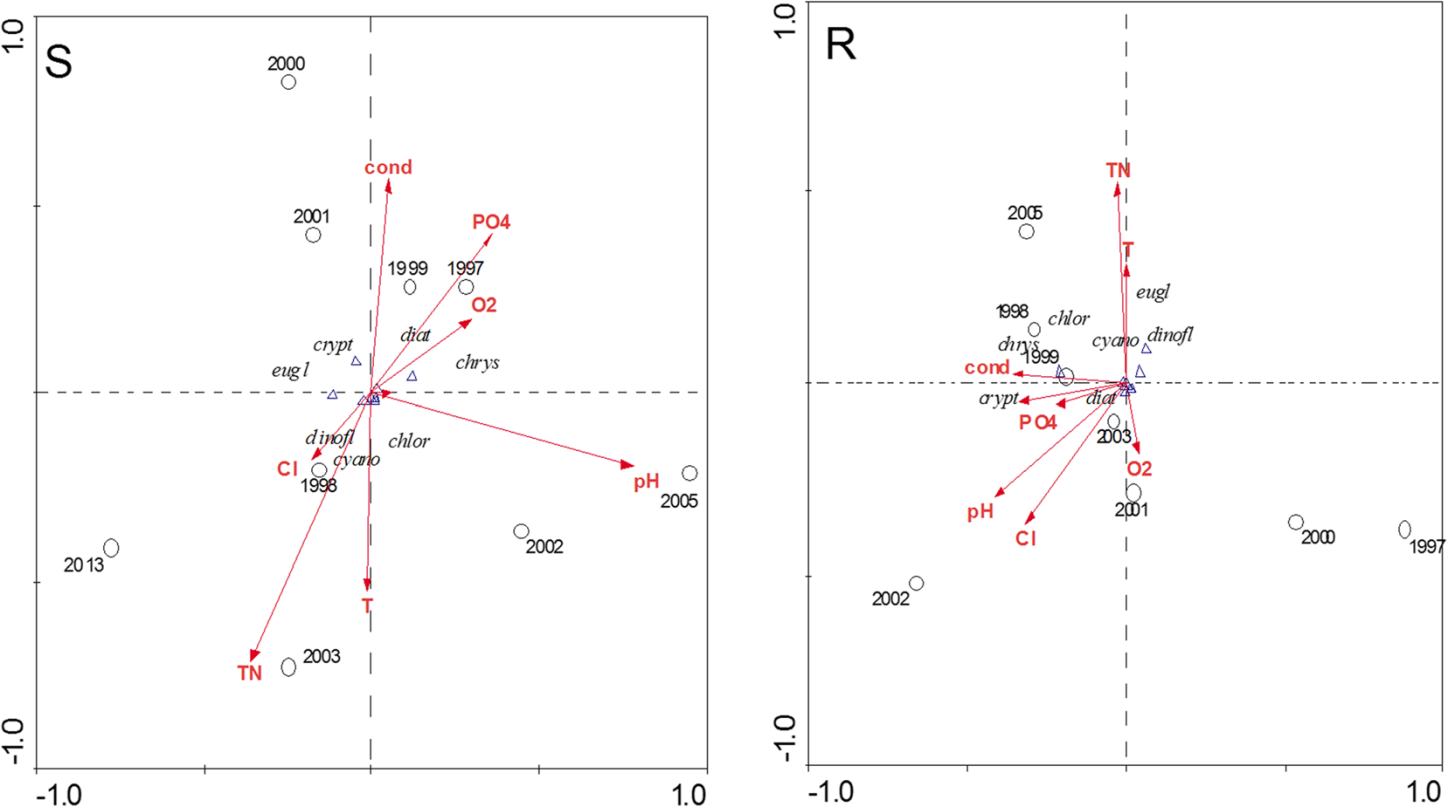
Biplot of the canonical correspondence analysis (CCA) showing the relationships between phytoplankton group abundance and physicochemical water parameters (*cyanob* cyanobacteria, *diat* diatoms, *chlor* chlorophytes, *dinofl* dinoflagellates, *chrys* chrysophytes, *crypt* cryptomonads, *eugl* euglenines) in the littoral zone (*S* separators, *R* macrophytes) in Lake Jeziorak Mały

CCA phytoplankton group analysis identified significant relationships between abundance and physicochemical variables in the studied years (Fig. 4). This employed a dataset of 50 samples, 7 phytoplankton groups, and 7 environmental variables, with the first axis accounting for 49.4 % of total (S) phytoplankton group variation and 44.4 % (R). Here, correlations were quite clear, cyanobacteria and dinoflagelates correlating with water temperature and TN, especially in 2003, diatoms with O_2_ and P-PO_4_ in 1997 and conductivity in 1999 at the separators. Moreover, at sites with macrophytes, also cyanobacteria, dinoflagellates, and euglenines correlated with water temperature and TN, especially in 2005, and diatoms, chrysophytes, and cryptomonads with conductivity (1999).

**Fig. 5:**
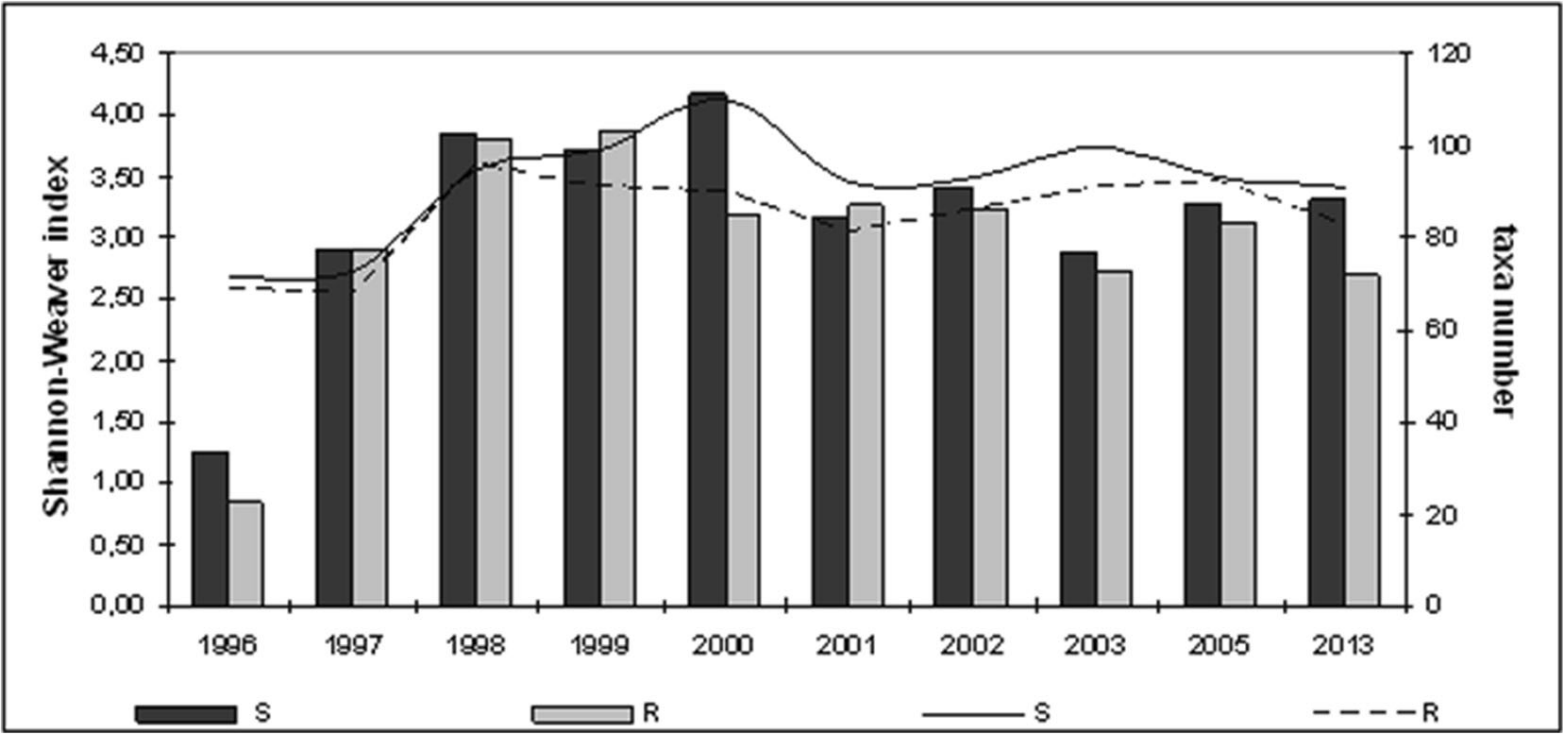
Species diversity and taxa number of phytoplankton in the littoral zone (*S* separators, *R* macrophytes) in Lake Jeziorak Mały in 1996–2003, 2005, and 2013

**Fig. 6:**
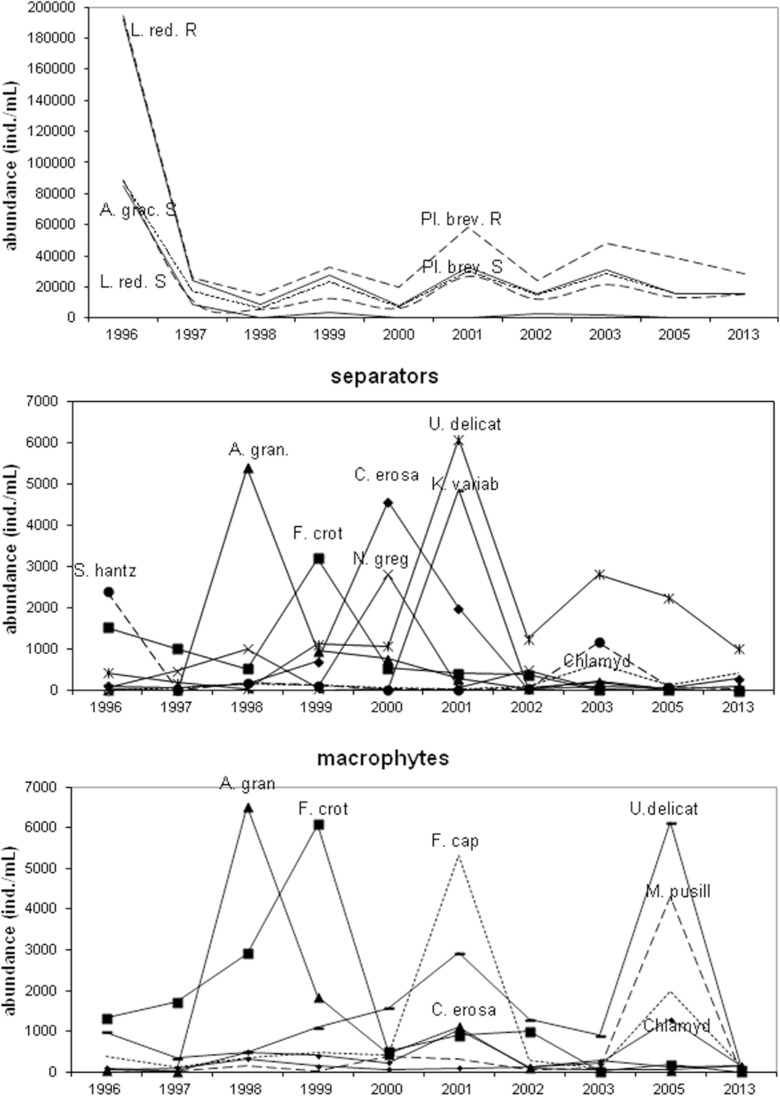
Abundance of dominant taxa of phytoplankton; (*A. grac Aphanizomenon gracile*, *L. red. Limnothrix redekei*, *Pl. brev. Planktolyngbya brevicellularis*, *S. hantz Stephanodiscus hantzschii*, *A. gran Aulacoseira granulata*, *F. crot Fragilaria crotonensis*, *C. erosa Cryptomonas erosa*, *N. greg Navicula gregaria*, *U. delicat Ulnaria delicatissima*, *K. variab Koliella variabilis*, *Chlamyd Chlamydomonas* sp., *F. cap Fragilaria capucina*, *M. pusill Micractinium pusillum*) in the littoral zone (*S* separators, *R* macrophytes) in Lake Jeziorak Mały in 1996–2003, 2005, and 2013

**Fig. 7:**
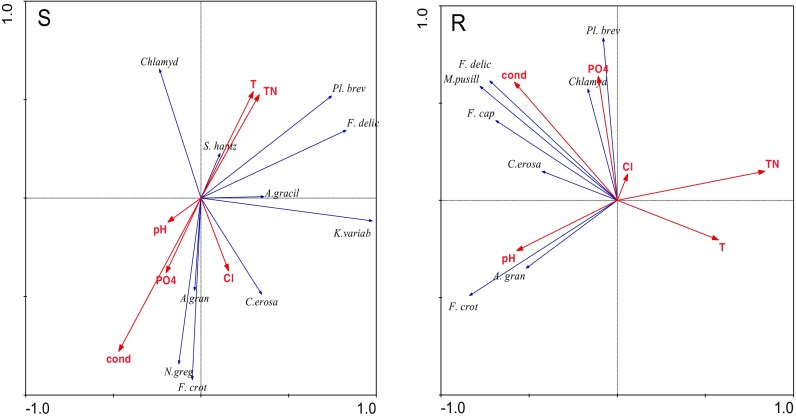
Biplot of the canonical correspondence analysis (RDA)-related phytoplankton dominant species abundance and physicochemical water parameters (*A. grac Aphanizomenon gracile*, *L. red Limnothrix redekei*, *Pl. brev Planktolyngbya brevicellularis*, *S. hantz Stephanodiscus hantzschii*, *A. gran Aulacoseira granulata*, *F. crot Fragilaria crotonensis*, *C. erosa Cryptomonas erosa*, *N. greg Navicula gregaria*, *U. delicat Ulnaria delicatissima*, *K. variab Koliella variabilis*, *Chlamyd Chlamydomonas* sp., *F. cap Fragilaria capucina*, *M. pusill Micractinium pusillum*) in the littoral zone (*S* separators, *R* macrophytes) in Lake Jeziorak Mały

The species diversity and taxa number increased in the studied period. Higher species richness was recorded at the separators (from 71 to 110 taxa) than at sites with macrophytes (from 69 to 92 taxa). The Shannon-Weaver index ranged from 1.26 to 4.18 bit ind.^−1^ (S) compared to from 0.84 to 3.87 bit ind.^−1^ (R) (Fig. 5). Changes in abundance of phytoplankton dominant taxa were noted in the studied years. This was especially noticeable in cyanobacteria. In 1996, cyanobacteria were dominated by *Limnothrix redekei* (Goor) Meffert with 94.83 % (S) and 96.47 % (R) and maximum abundance at 85,035 ind. mL^−1^ (S) and 103,280 ind. mL^−1^ (R). In the following years, there was decreased proportion and abundance of this species and increased *Planktolyngbya brevicellularis* Cronberg & Komárek (S—84.24 % in 1998 and 72.27 % in 2013 and R—81.05 % in 2013) and *Aphanizomenon gracile* Lemm. (S—44.72 % in 1999). *P. brevicellularis* has reached the maximum abundance of 26,460 ind. mL^−1^ (S) and 25,690 ind. mL^−1^ (R) in 2001. Diatom abundance was dominated by the following species at the separators: *Stephanodiscus hantzschii* Grunow (in Cleve & Grunow) (1996), *Aulacoseira granulata* (Ehrenb.) Simonsen (1998), *Fragilaria crotonensis* Kitton (1999), *Navicula gregaria* Donkin (2000), and *Ulnaria delicatissima* (W.Smith) Aboal & P.C.Silva (2001). In addition, sites with macrophytes were dominated by *A. granulata* (1998), *F. crotonensis* (1999), *Fragilaria capucina* Desm. (2001), and *U. delicatissima* (2005), and in 2013, these were accompanied by *F. capucina* and *Ulnaria acus* (Kützing) Aboal. Chlorophytes were dominated by *Chlamydomonas* spp. (S—max. 1180 ind. mL^−1^ in 2003 and R—max. 1280 ind. mL^−1^ in 2005), and also by *Koliella variabilis* (Nygaard) Hindák in 2001 at the separators and *Micractinium pusillum* Fres. in 2005 at sites with macrophytes. The following dominant species also occurred: (1) cryptomonads were dominated by *Cryptomonas erosa* Ehrenb. (S—max. 4560 ind. mL^−1^ and R—1035 ind. mL^−1^ in 2001); (2) dinoflagellates by *Ceratium hirundinella* (O.F.Müller) Dujardin in 1996–1997 (S) and 1996 and 2001 (R) and *Parvodinium inconspicuum* (Lemmermann) S. Carty in the remaining years (Fig. 6); (3) chrysophytes by genera *Mallomonas* sp. and *Dinobryon* sp.; and (4) euglenines by genus *Trachelomonas* sp. RDA analysis identified significant relationships between abundance of phytoplankton dominant species and physicochemical properties (Fig. 7). *P. brevicellularis* correlated with water temperature and TN (*r* = 0.72 and *r* = 0.69), and *F. crotonensis* and *N. gregaria* with conductivity (*r* = 0.76 and *r* = 0.72) at the separators. Moreover, at site with macrophytes, *F. delicatissima*, *F. capucina*, and *M. pusillum* correlated with conductivity (at *r* = 0.82, *r* = 0.72, and *r* = 0.75); *P. brevicellularis* and *Chlamydomonas* spp. with P-PO_4_; and *A. gracile* and *F. crotonensis* with pH. In addition here, negative correlation was established between *A. gracile* and P-PO_4_ (*r* = −0.73), *F. crotonensis* and TN (*r* = −0.69), and *M. pusillum* and water temperature (*r* = −0.74).

### Summer phytoplankton in the pelagial

**Fig. 8:**
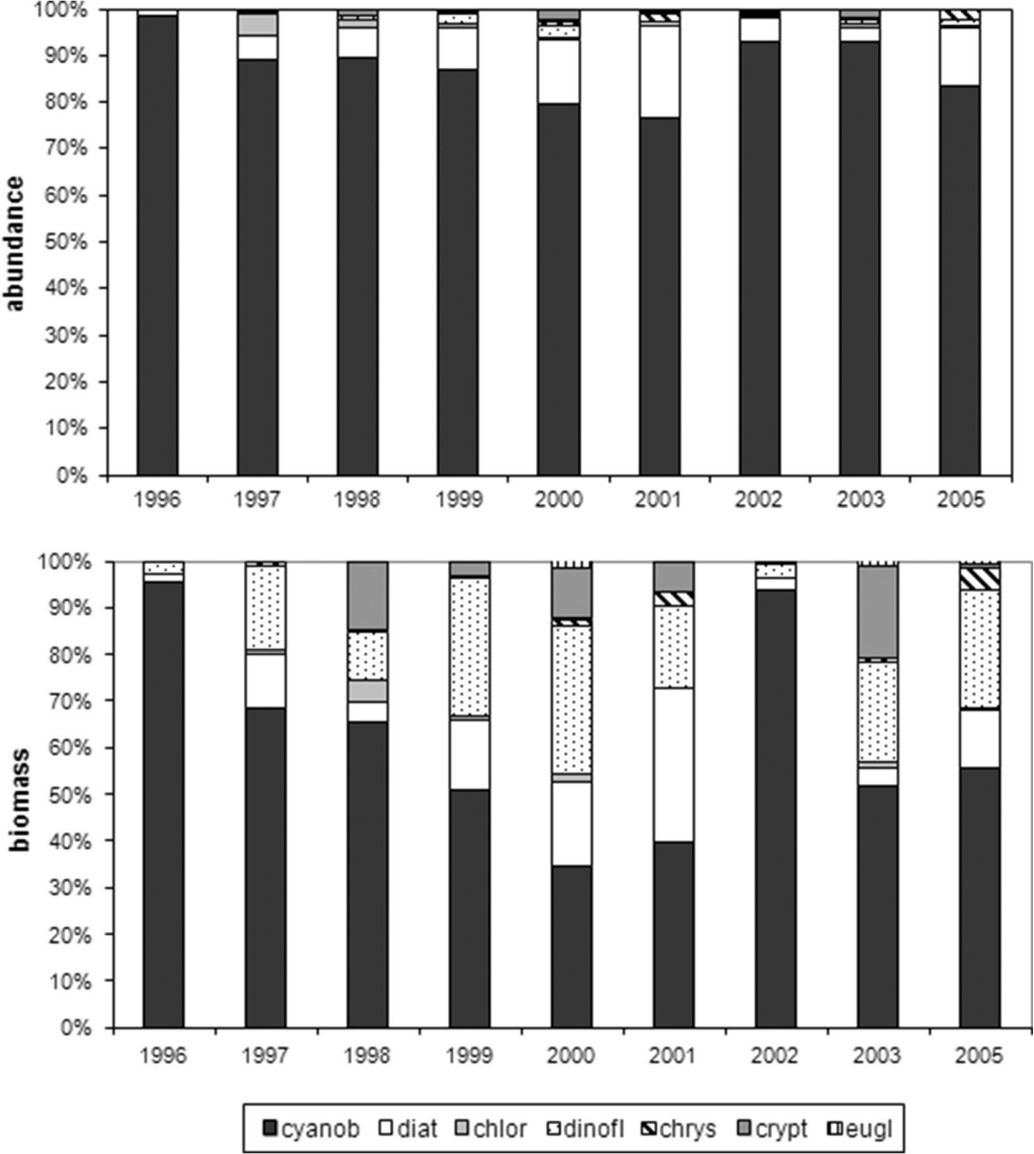
Structure of phytoplankton (*cyanob* cyanobacteria, *diat* diatoms, *chlor* chlorophytes, *dinofl* dinoflagellates, *chrys* chrysophytes, *krypt* cryptomonads, *eugl* euglenines) in the pelagial in Lake Jeziorak Mały in 1996–2003 and 2005

This study revealed pelagial summer phytoplankton abundance and biomass domination by cyanobacteria in 1996–2003 and 2005. Maximum algal proportion was 98.69 % in 1996, followed by fluctuations to 76.67 % in 1997–2003 and 92.82 % in 2003 and final dip to 63.80 % in 2005. Although higher diatom and chlorophyte abundance registered 33.72 % in 2005 and 4.82 % in 1997, the remaining phytoplankton group quota did not exceed 4 %. Meanwhile, the cyanobacterial proportion of total phytoplankton biomass decreased from 95.60 % in 1996 to 34.72 % in 2000 with following recovery to 55.43 % in 2005. This was accompanied by increased of diatoms (30.32 % in 2005), dinoflagellates (31.94 % in 2000), cryptomonads (19.81 % in 2003), and chrysophytes (4.57 % in 2005) (Fig. 8). Maximum cyanobacterial abundance was attained in summer 1996 at 330,000 ind. mL^−1^, with rapid loss in 1997 followed by increase in 1998 and 2002 and then reducing to 11,340 ind. mL^−1^ in 2005.

**Fig. 9:**
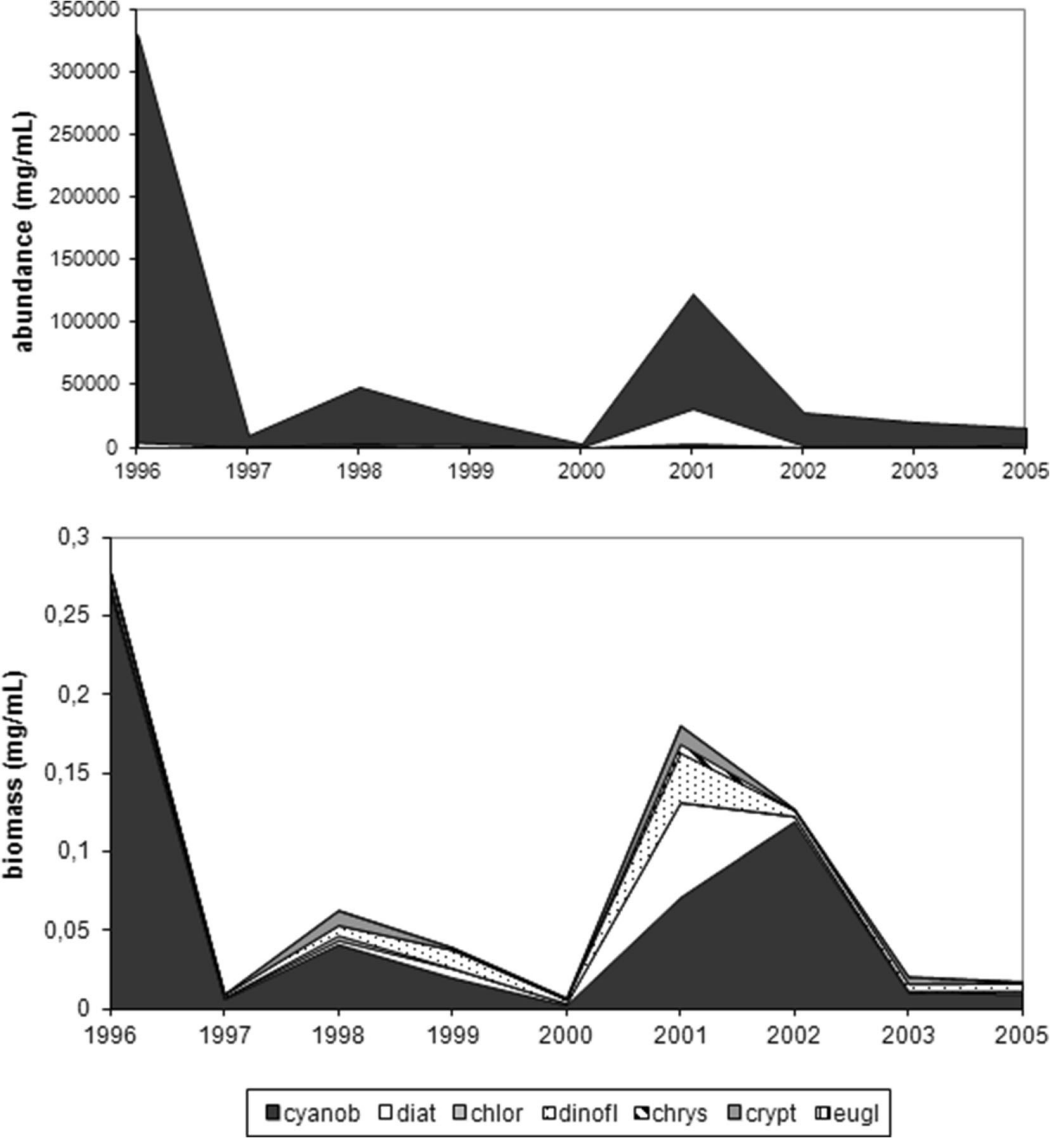
Dynamics of phytoplankton abundance and biomass (*cyanob* cyanobacteria, *diat* diatoms, *chlor* chlorophytes, *dinofl* dinoflagellates, *chrys* chrysophytes, *crypt* cryptomonads, *eugl* euglenines) in the pelagial in Lake Jeziorak Mały (means from June to August in 1996–2003 and 2005)

There was also gain in diatom share to its greatest 3210 ind. mL^−1^ level in 2001. Similar changes noted in the phytoplankton group biomass with massive collapse of cyanobacteria biomass in 1997, then considerable elevation by 2002 until further decline in 2005. These changes were accompanied by significant upward trend in diatom, dinoflagellate, and cryptomonad biomass (Fig. 9).

Similar to occurrences observed in the littoral zone, exchanges in dominant taxa were also recorded in the pelagial, especially the change in cyanobacteria from *L. redekei* (96.35 % in 1996) to *P. brevicellularis* (97.75 % in 2005). In addition, (1) diatoms were dominated by the following species: *Asterionella formosa* Hassall in 1996, *F. crotonensis* in 1997–1999, and *U. delicatissima* in the remaining years; (2) chlorophyte dominants were *Chlamydomonas* spp. (1996–1998 and 2003), *Desmodesmus communis* E. Hegewald (1999), *Monoraphidium griffithii* (Berkeley) Komárková-Legnerová (2000), *Tetraedron minimum* (A. Braun) Hansgirg (2001 and 2003), and *Golenkinia radiata* Chodat (2005); (3) dinoflagellates were dominated by *C. hirundinella* in 1996 and then by *P. inconspicuum*; (4) the chrysophytes dominant was genus *Dinobryon* sp.; (5) cryptomonads were dominated by *C. erosa*; and (6) euglenines by genus *Trachelomonas* sp.

## Discussion

Lake Jeziorak Mały is very susceptible to eutrophication, with developments similar to those observed in shallow polimictic urban lakes by Guzkowska and Gasse (1996), Mischke and Nixdorf (2003), and Kangro et al. (2005). Phytoplankton studies and water quality classified this lake as politrophic until the 1997 introduction of lamella separators, stone accumulation in the littoral zone and a fountain-based water aeration system in the pelagial (Niewolak 1974; Spodniewska 1986; Zębek 2009). Between 1960 and 1990, the water had low oxygen in the pelagial, with oxygen deficit near the lake bed often causing fish death (Jankowski 1966). Cyanobacterial blooms of *Anabaena*, *Aphanizomenon*, and *Microcystis* genera were discovered in the 1968 summer season, with abundant *Oscillatoria* and *Asterionella* in that autumn (Niewolak 1974) and mass *Planktothrix agardhii* Gom. cyanobacterial growths in 1978 (Spodniewska 1986). Despite municipal sewage management, stormwaters are often the source of water pollution in lakes such as urban Jeziorak Mały, where water quality changes can be rapid and extreme as inflows vary in quantity, chemistry, and seasonality. This is exacerbated by unpredictable occurrences such as building construction and road salting in the surrounding catchments (Guzkowska and Gasse 1990). Storm effluents from the catchment contribute to dramatic water disturbance at the separators, with subsequent changes and higher water fertility. Although polluted stormwater from the catchment area significantly affected lake water chemistry by enhancing nutrient concentrations, it also contained elevated Pb, Cu, Zn, Cd heavy metals and other chemical elements (Szpakowska et al. 2014; Sapek 2014; Zębek and Szwejkowska 2014). In addition to sewage inflow prevention, stormwater pretreatment is also vital. As in other renovated urban lakes, Lake Jeziorak Mały utilized separators to pretreat stormwater and remove organic substances, petroleum compounds, and silt and sand (Guzkowska and Gasse 1990; Galloway et al. 2003; Hsieh and Davis 2005; Taylor et al. 2005). This installation greatly altered environmental conditions compared to sites with macrophytes, initiating lower mean water temperature and pH, higher conductivity and chlorides, and highest local orthophosphate and total nitrogen concentrations; consequently, phytoplankton emerged as a reliable trophic indicator.

Separator function changed Lake Jeziorak Mały phytoplankton structure, abundance, and biomass and also altered species diversity, with dominant taxa observed in 1997–2003, 2005, and 2013. The greatest reduction in the cyanobacterial proportion of total phytoplankton, abundance, and biomass in this period was accompanied by higher ratios, number and biomass of diatom, cryptomonads, dinoflagellates, and chlorophytes. This altered phytoplankton growth was more dynamic and rapid at the separators than at sites with macrophytes, with resultant lower cyanobacteria percentage in total phytoplankton abundance. Although storm effluent greatly disturbed cyanobacterial growth, especially at maximum precipitation levels over 100 mm (Zębek and Szwejkowska 2014). Statistical analysis determined that water temperature was very important in stimulating cyanobacteria and dinoflagellates, and further, the elevated amount of oxygen was due to the rapid growth of diatoms (S). The unique phytoplankton environmental requirements confirm stormwater influence. While (S) and (R) locations experienced different conditions, the following diverse effects were highlighted by statistically significant correlations and CCA. High water temperatures and total nitrogen concentrations favored cynaobacterial and dinoflagellate growth especially in 2003, with mean water temperatures above 18 °C at the separators and in 2005 with 19 °C at sites with macrophytes. Moreover, mass diatoms development occurred at high mean P-PO_4_ and oxygen content especially directly after the separator installation in the littoral zone in 1997. In addition, diatom, chrysophyte, and cryptomonad growth was regulated by conductivity at the sites with macrophytes especially in 1999. The significant differences and lower dynamics in phytoplankton group abundance and biomass are most likely due to the limited influence of separator-treated stormwater exerted at the sites with macrophytes (Zębek 2015a). This study highlights that stormwater treatment is essential to preclude organic matter, including nutrients, from urban lakes. Moreover, existing stormwater management procedures must be updated to supplement separator installation and further limit excessive nutrient concentrations after rainfall.

Rapid changes in physical and chemical water parameters and intensive mineralization favored cyanobacteria, diatom, chrysophyte, cryptomonad, and euglenin P-PO_4_ nutrient uptake from the waters, especially supporting by negatively correlation between *A. gracile* and P-PO_4_ and *F. crotonensis* and TN in the littoral zone. Here, periphyton can be gainfully employed in biomanipulation, competing with phytoplankton for nutrients and reducing their biomass and bloom frequency (Hansson 1990; Danilov and Ekuland 2001). Artificial substrata are also submerged in the pelagial for better lake management (Jöbgen et al. 2004), with stone accumulation in Lake Jeziorak Mały encouraging periphyton growth and nutrient uptake. This provides a very simple economical method for resettling nutrient-rich water bodies by creating new habitats for organisms absorbing excessive nutrients.

One important problem remaining for urban shallow lakes is oxygen deficit in bottom layers caused by organic matter accumulation on the lake bed. Fountain-based aeration to improve lake oxygenation provides the best remedy (Visser et al. 1996; Klapper 2003; Hanson and Austin 2012; Moore et al. 2012). All instituted restoration measures in Lake Jeziorak Mały, included fountain installation, could contribute to altered environmental conditions for summer phytoplankton development in the pelagial. Increased water transparency and oxygenation, lower pH and conductivity and reduced orthophosphates at the lake bottom were all obvious during this study period, as also were the significant decline in cyanobacterial species distribution, abundance, and biomass from 98 to 70 %. The fountain’s successful water mixing disrupted cyanobacterial growth and stimulated diatom and chlorophyte abundance and diatom, dinoflagellate, cryptomonad, and chrysophyte biomass (Zębek 2015b). The dramatic change in phytoplankton and water quality noted directly after the start of Lake Jeziorak Mały restoration was comparable to results in Visser et al. (1996) and Jeppesen et al. (2007). These authors reported also P-PO_4_ reduction and registered lower P-PO_4_ content near the fountain lake bottom. Fountain initiated water mixing intensified sediment mineralization and nutrient resuspension, rising nutrient concentrations at the lake bottom and boosting phytoplankton uptake. Subsequent water phosphate reduction is especially important in nutrient-rich shallow lakes to limit excessive cyanobacteria growth and ensure appropriate water quality. While positive effects from this complex process may only be obvious in the long term, precaution must also be taken to prevent aeration and water-mixing promoting extreme lake-bottom disturbance and secondary eutrophication. Studies of cyanobacteria response to the fountain-based aeration confirmed seasonal dynamic disturbances without abundance and biomass limitation at the fountain (Zębek 2014).

Lake trophic conditions determine species diversity. Lake Jeziorak Mały is comparable to other restored lakes (Jeppesen et al. 2007) with significant increment in species diversity and taxa number following restoration measures. Dominant species changes from politrophic to eutrophic conditions were obvious, for example, in the polytrophic *Limnothrix redekei* replacement by eutrophic *Planktolyngbya brevicellularis* (Reynolds 1984; Cronberg and Komarek 1994; Jöbgen et al. 2004; Zębek 2006). In this study, *P. brevicellularis* growth was mainly stimulated by water temperature and TN at the separators and P-PO_4_ at sites with macrophytes. Additional examples include *Stephanodiscus hantzschii* supplemented by *Aulacoseira granulata* (S) and *F. crotonensis* and *U. delicatissima*, *Fragilaria* sp. diatoms at both (S) and (M) in meso and eutrophic lakes (Reynolds 1984; Bucka 1989), and displacement of *Asterionella formosa* by eutrophically typical diatoms *F. crotonensis* and *U. delicatissima* in the pelagial. In the littoral zone of Lake Jeziorak Mały, *F. crotonensis* and *N. gregaria* growth was regulated by water conductivity at the separators, similarly to *U. delicatissima*, *F. capucina*, and *M. pusillum* at sites with macrophytes. Other eutrophic phytoplankton taxa rose in abundance after 1996, with (a) chlorophytes genus *Chlamydomonas* spp., *Koliella variabilis*, and *M. pusillum* in the littoral zone superseding *Chlamydomonas* spp. *Desmodesmus communis* and *Monoraphidium griffithii* (Schrenk-Bergt et al. 2004) and (b) the presence of dinoflagellates such as *Parvodinium inconspicuum* and *Ceratium hirundinella* which often inhabit nutrient-rich waters (Bucka 1989; Gligora et al. 2003). In this study, *Chlamydomonas* spp. favored high orthophosphates and *M. pusillum* low water temperature. These changes in phytoplankton dynamics and physicochemical properties and environmental requirements of dominant species highlight beneficial response to restoration.

## Conclusion

The present study provides significant data on the great benefits derived from shallow urban lake restoration. Lake Jeziorak Mały separator and fountain installation initiated transformation from polytrophic to eutrophic conditions, consequently altering phytoplankton structure and dynamics. These measures induced effective dynamics with rapid progression in the littoral zone, especially the ameliorated phytoplankton dynamics and environmental conditions more concentrated at separators than at macrophytes. We determined stabilization in 2013 where phytoplankton recovered its average levels. Moreover, this study also clearly demonstrates that the restorative procedures, including the fountain-based aeration, induced changes in the summer phytoplankton structure and physicochemical water parameters. This encouraged also phytoplankton nutrient uptake and increased P-PO_4_ in lake bed layers, with lower summer cyanobacterial percentage and high water transparency. Despite the noticeable improvement of water quality, the phosphorus concentrations in the lake are still high and greater improvements in water quality can be achieved by using an additional method of restoration, e.g., inactivation of phosphorus in the water column. Understanding phytoplankton-environment relationships is paramount in lake management strategies. Here, Lake Jeziorak Mały long-term phytoplankton succession confirms appropriate response to protective-restoration measures necessary in all urban lake management. Moreover, slower phytoplankton response in the pelagial highlights the significant littoral influence on lake trophic state. This zone therefore requires greater legal protection on an international scale. In conclusion, our study highlights that these restorative effects of limited cyanobacteria growth and revised physicochemical water parameters require continuation and update, and these are equally essential in all shallow urban lake management.
